# Molecular Dynamics Studies of Ordered and Disordered Lanthanum Cerate: Thermodynamic Stability, Heat Capacity, and Oxygen Diffusivity

**DOI:** 10.1002/cphc.70614

**Published:** 2026-11-11

**Authors:** Bianca Dißmann, Roger A. De Souza

**Affiliations:** ^1^ Institute of Physical Chemistry RWTH Aachen University Aachen Germany

**Keywords:** ceramics, defect fluorite, molecular dynamics, oxygen diffusion, pyrochlore

## Abstract

Oxides with the formula *A*_2_ *B*_2_O_7_ may crystallize either as ordered pyrochlores or as oxygen‐deficient defect fluorites. Lanthanum cerate is a material for which both structures have been proposed in the literature. In this study, molecular dynamics (MD) simulations with many‐body potentials are used to examine as a function of temperature T the relative stability of the two phases, as well as of intermediate phases with varying degrees of cation disorder χ. The simulations also yield as a function of T and χ the isobaric heat capacity $C_{p}$ and the oxygen tracer diffusivity $D_{O}^{*}$. Taking the calculated cell energies and considering in addition configurational entropy, we find that defect fluorite structures are thermodynamically favored for lanthanum cerate. $C_{p}(T)$ data for ordered structures exhibit an anomalous increase at higher temperatures, indicative of anti‐Frenkel disorder. Converting calculated oxygen tracer diffusion coefficients into ionic conductivities and comparing with reported experimental data, we find good agreement, especially concerning the activation enthalpy, with data for the defect fluorite structure.

## Graphical abstract

Fully ordered (pyrochlore) and fully disordered (defect fluorite) structures of lanthanum cerate, as well as of intermediate disordered structures, are studied at finite temperatures through atomistic simulations. Disordered structures are found to be thermodynamically more stable than the ordered structure. Isobaric heat capacity and the oxygen tracer diffusivity are shown to be sensitive in different ways to disorder.

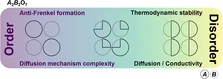

## 1 Introduction

The crystal structure of the pseudobinary compound lanthanum cerate (LCO) is a subject of much debate. Two closely related structures have been proposed, the ordered pyrochlore structure (OP, *A*_2_ *B*_2_O_7_, space group $Fd\bar{3}m$) and the disordered defect fluorite structure (DF, (*A*_0.5_ *B*_0.5_)O_1.75_, space group $Fm\bar{3}m$). The OP structure is characterized by two cation sites (A in 16d and B in 16c Wyckoff positions), by two oxygen sites (48f and 8b), and by an empty oxygen site (8a). The DF structure is characterized by a single‐cation site (4a), on which A and B cations are distributed at random, and by a single‐anion site (8c), whose eight positions are occupied at random with seven oxide ions. The two structure types are illustrated in Figure 1.

**FIGURE 1:**
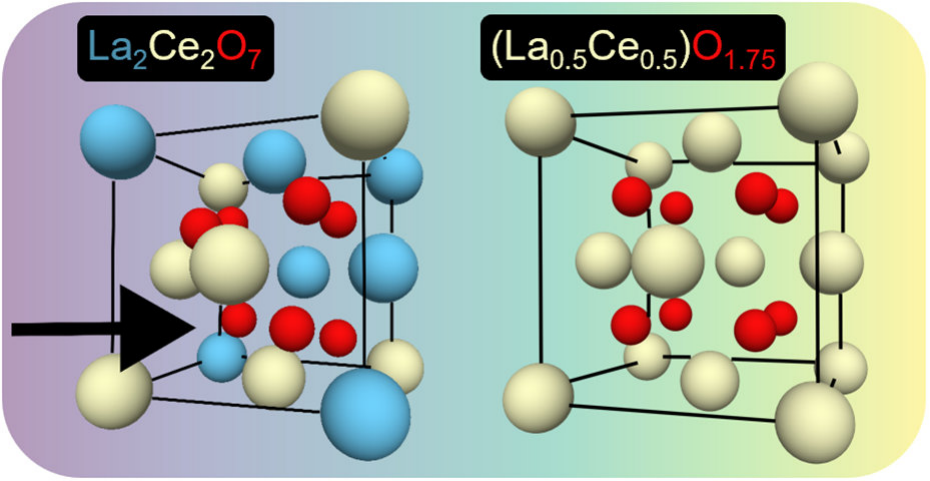
(Left) Ordered pyrochlore (OP) structure with ordered cations and anions, and an (empty) interstitial site (indicated by the arrow). (Right) Defect fluorite (DF) structure with two cations randomly distributed over the one cation site and anions occupying at random seven out of eight positions of the single‐anion site [1].

Experimental studies tend to favor the DF structure. From the earliest study [2] onwards, X‐ray diffraction (XRD) patterns often, but not always, lack pyrochlore‐specific reflections [3, 13], and consequently there is a preference for the DF structure. In those cases where characteristic pyrochlore reflections are found (e.g., [13]), their intensities are much lower and much broader than those observed in a pattern of a clearly identifiable pyrochlore structure, such as La_2_Zr_2_O_7_. Neutron powder diffraction (NPD), being more sensitive to oxygen, should provide a clearer answer, but three NPD studies [12, ‍14, ‍15] concluded that the DF structure was present, while a fourth favored the OP structure [16]. Examining X‐ray pair distribution functions (XPDF) for LCO, Mowe et al. [13] found the pyrochlore structure to describe best the data, but for larger interatomic distances, a description with the fluorite structure was also possible. Raman spectroscopy studies also do not lead to a clear preference for one structure‐type but often conclude that mixtures of different types are present [9, 10, 13]. Pyrochlore microdomain ordering can be found, for example, in other DF‐like structured compounds [17]. Overall, it appears that the structure‐type favored in a particular study depends strongly on the method used and if the method probes short‐range or long‐range ordering.

Computational studies present a more uniform picture: they all conclude in favor of the OP structure. Minervini et al. [18] performed atomistic simulations of a wide variety of *A*_2_ *B*_2_O_7_ pyrochlore oxides, predicting that LCO was stable as an OP but only just, being on the “cusp of stability”. Density functional theory (DFT) calculations [14, 19, 20, 21] have a preference for the OP structure (at least for the cation lattice). Disordering the anion sublattice is found to have a significant positive impact on stability in contrast to the cation lattice. Vanpoucke et al. [19] also generated XRD patterns and found that the peak positions agreed with experimental data for both structures, but the intensities were in better agreement with those for the pyrochlore structure.

In summary, the experimental and computational methods do not agree as to the structure type of LCO. Computational studies lean toward the OP type, whereas most experimental methods favor the DF type (at least for those methods that probe long‐range order). Still, based on different experimental methods, both structure types could be present at the same time because of differences in short‐ and long‐range order or different ordering on the cation and anion lattices.

Pyrochlore oxides have attracted attention for their various properties. Oxide‐ion transport in pyrochlore‐based systems in general has attracted attention for many years, both experimentally [13, 22, 23, 24, 25, 26, 27, 28, 29, 30, 31, 32] and computationally [33, 34, 35, 36, 37, 38, 39]. Wilde and Catlow [33] performed static atomistic calculations of selected zirconate and titanate pyrochlores and confirmed earlier considerations [26, 36] that oxygen diffusion occurs predominantly by vacancy migration between interconnected 48f sites. Perriot and Uberuaga [37] used molecular dynamics (MD) simulations to investigate oxygen diffusion in Gd_2_Zr_2_O_7_ and Gd_2_Ti_2_O_7_ pyrochlores, focusing on the effect of cation sublattice disorder on oxygen diffusion. The simulations indicated that cation disorder generally enhances diffusion, on account of oxygen vacancies being trapped in the ordered structure, and that disordered materials contain more defects (here: oxygen vacancies). Additional oxygen vacancies (on 48f sites) or oxygen interstitials (on 8a sites) strongly increase the diffusivity. Defects can, however, be trapped by cation antisites, especially in more ordered structures. The diffusion‐enhancing factor of cation disorder can also be found for other pyrochlore compounds [40]. A connection between disorder on the anion lattice as well as the cation lattice and higher diffusion coefficients can also be found in experimental studies with neutron scattering through doping series [27, 41, 42].

For all these studies, it has to be kept in mind that LCO was not investigated, and predictions based on similar compounds have to be handled carefully. This is especially the case since LCO is a more complicated system, with oxide ions being only one type of mobile species. In wet atmospheres, LCO can dissolve water, forming hydroxide ions [10, 13, 22, 24, 43], and consequently, protons and/or hydroxide ions may be mobile in LCO—in addition to mobile electronic charge carriers. In this study, we focus on oxygen diffusion.

In this study, we employed MD simulations with many‐body potentials to calculate various properties of the LCO system, from the ordered to the disordered structure. We aimed, first, to resolve the discrepancy between experimental and computational studies concerning the relative phase stability and, second, to determine how sensitive macroscopic properties, such as heat capacity and oxygen tracer diffusivity, are to disorder in the system.

We employed the many‐body potentials derived by Cooper, Rushton, and Grimes (CRG) [44] for two main reasons. They are robust at the high temperatures generally employed in MD simulations. And they have been successfully applied to modeling point defects and their diffusion in fluorite‐structured ${\mathrm{CeO}}_{2}$ [45, 46] and pyrochlore‐structured La_2_Zr_2_O_7_ [34].

## 2 Results and Discussion

### 2.1 Phase Stability: OP Versus DF

First, we consider the reaction of the OP phase La_2_Ce_2_O_7_ to the DF phase (La_0.5_Ce_0.5_)O_1.75_

(1) $${\mathrm{La}}_{2}{\mathrm{Ce}}_{2}O_{7}⇌4\;({\mathrm{La}}_{0.5}{\mathrm{Ce}}_{0.5})O_{1.75}$$

with transition energy

(2) $$\Delta_{\mathrm{trs}}E(T)=E_{\mathrm{tot}}^{\mathrm{DF}}(T)-E_{\mathrm{tot}}^{\mathrm{OP}}(T)$$

Values of $E_{\mathrm{tot}}^{\mathrm{OP}}(T)$ were obtained as the average from three MD simulations, each with a different starting seed; the same OP simulation cell was used in all three cases. The values of $E_{\mathrm{tot}}^{\mathrm{DF}}(T)$ were obtained from MD simulations of three different cells, each with a different random order, and averaging over their energies. Each DF simulation cell was obtained from a CeO_2_ supercell from which half of the Ce cations were randomly substituted with La cations and from which one eighth of all oxide ions were randomly removed; in addition, a Metropolis Monte Carlo (MMC) energy minimization was carried out, swapping oxide ions and oxygen vacancies, in order to accelerate the equilibration of the anion sublattice [47]. The results are plotted in Figure 2a.

**FIGURE 2:**
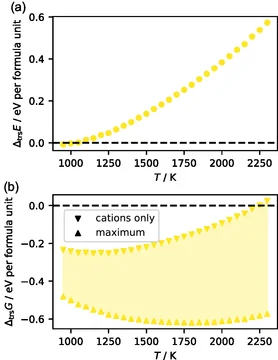
Transition from the ordered pyrochlore La_2_Ce_2_O_7_ to the defect fluorite (La_0.5_Ce_0.5_)O_1.75_: (a) transition energy $\Delta_{\mathrm{trs}}E$ obtained from MD simulations; (b) Gibbs transition energy $\Delta_{\mathrm{trs}}G$ obtained from $\Delta_{\mathrm{trs}}E$ and contributions from configurational entropy. The shaded area refers to the range of $\Delta_{\mathrm{trs}}G$ for intermediate degrees of oxygen vacancy ordering. The dashed black line indicates a (Gibbs) energy difference of zero.

$\Delta_{\mathrm{trs}}E$ is seen to vary from just below 0 eV at *T* = 950 K to almost 0.6 eV at *T* = 2300 K (no data are available for even higher temperatures, as DF simulation cells were unstable at *T* > 2300 K). The positive value of $\Delta_{\mathrm{trs}}E$ would appear to indicate that the OP structure is more stable than the DF structure over most of the given temperature range (except for *T* < 1050 K).

Strictly speaking, in order to determine the relative phase stability at given temperature and pressure, one should use the Gibbs transition energy $\Delta_{\mathrm{trs}}G$ rather than $\Delta_{\mathrm{trs}}E$, since *G* is the relevant thermodynamic potential. $\Delta_{\mathrm{trs}}G$ contains contributions from entropy ($-T\Delta_{\mathrm{trs}}S$), and in the present case we only considered changes in configurational entropy $S_{\mathrm{cfg}}$, ignoring changes in vibrational entropy $S_{\mathrm{vib}}$, so that $\Delta_{\mathrm{trs}}S\approx \Delta_{\mathrm{cfg}}S$. Equating the transition enthalpy $\Delta_{\mathrm{trs}}H$ with the transition energy $\Delta_{\mathrm{trs}}E$ (due to p=0 in the simulations), we thus calculated the Gibbs free energy of the phase transition according to

(3) $$\Delta_{\mathrm{trs}}G=\Delta_{\mathrm{trs}}H-T\Delta_{\mathrm{cfg}}S$$

For the DF structure, $S_{\mathrm{cfg}}$ for the (La,Ce) sublattice is that of a completely random system (as this is how the simulation cells were generated), and hence it is given by

(4) $$\begin{array}{cc} S_{\mathrm{cfg},\mathrm{cat}} & =-k_{B}\cdot N_{s,\mathrm{cat}}\cdot \left(x_{\mathrm{La}}\ln\;x_{\mathrm{La}}+x_{\mathrm{Ce}}\ln\;x_{\mathrm{Ce}}\right)\\ & =-k_{B}\cdot 4\cdot \left(\frac{1}{2}\ln\;\frac{1}{2}+\frac{1}{2}\ln\;\frac{1}{2}\right) \end{array}$$

where $N_{s,\mathrm{cat}}$ is the number of cation sites per DF unit cell, and $x_{j}$ is the site fraction of species j. A less trivial case is that of the anion sublattice in the DF phase, where the configuration entropy of the simulation cell lies between that of a random distribution

(5) $$\begin{array}{cc} S_{\mathrm{cfg},\mathrm{an}} & =-k_{B}\cdot N_{s,\mathrm{an}}\cdot \left(x_{O}\ln\;x_{O}+x_{v}\ln\;x_{v}\right)\\ & =-k_{B}\cdot 8\cdot \left(\frac{7}{8}\ln\;\frac{7}{8}+\frac{1}{8}\ln\;\frac{1}{8}\right). \end{array}$$

and an ordered distribution. The initial random distribution was, namely, modified substantially during the MMC run, as indicated by the comparably high amount of accepted exchanges and the comparably large decrease in system energy (not shown). Since $S_{\mathrm{cfg},\mathrm{an}}$ cannot be easily determined, we consider two extreme cases for the DF phase: the entropy for the fully random cation sublattice, and the entropy for fully random cation and anion sublattices.

The configurational entropy for the cation sublattice in the OP structure is zero because there is only one configuration possible and there is no change in the cation distribution during the simulation. For the anion lattice, we assume reasonably an $S_{\mathrm{cfg}}$ of zero: At the higher temperatures of our MD simulations, some oxide ions will reside on interstitial sites, leaving vacancies behind (see Section 2.2), but the small amount of disorder produces only a small contribution to the configurational entropy, and this is negligible compared with the terms for the DF phase.

In Figure 2b the transition free enthalpy $\Delta_{\mathrm{trs}}G$ is shown for both extremes. We see that including only $\Delta_{\mathrm{cfg}}S_{\mathrm{cat}}$ (and thus setting $\Delta_{\mathrm{cfg}}S_{\mathrm{an}}/k_{B}=0$) is already sufficient to shift the stability curve in Figure 2a for almost the whole temperature range below zero, favoring the DF phase. If we now also include the entropy contribution from a fully disordered anion sublattice ($\Delta_{\mathrm{cfg}}S_{\mathrm{an}}/k_{B}\neq 0$), we find that the curve is shifted to even lower values, favoring the DF phase even more strongly. The true $\Delta_{\mathrm{trs}}G$ of these simulations, as noted above, will lie somewhere between the two curves, and the general trend with increasing temperature will be toward more negative $\Delta_{\mathrm{trs}}G$, owing to the increasing amount of disorder on the oxygen sublattice of the simulated DF phase (decreasing tendency of oxygen vacancies in the DF phase to adopt ordered configurations). Hence, by including configuration entropy, we predict that, for the range of temperatures considered, the DF phase is thermodynamically more stable than the OP phase.

### 2.2 Anti‐Frenkel Disorder: OP Versus DF

For the OP phase, given the presence of the empty 8a site (which is sometimes termed a structural oxygen‐vacancy, but from a defect perspective is more correctly considered to be an interstitial site), anti‐Frenkel disorder may be expected to generate oxygen interstitials (on the 8a site) and oxygen vacancies (on the 48f site) at high temperatures

(6) $$O_{O,48f}^{\times }+v_{i,8a}^{\times }⇌v_{O,48f}^{••}+O_{i,8a}^{\text{''}}$$

The thermodynamics of this defect reaction can be determined quantitatively by examining the isobaric heat capacity $C_{p}$. An anomalous increase in $C_{p}$ is expected at higher temperatures, as more and more anti‐Frenkel defects are formed, which increase the system’s enthalpy. The behavior of $C_{p}$ as a function of temperature can thus be described as [34, 48, 49, 50, 51]

(7) $$C_{p}(T)=C_{p}^{0}(T)+\Delta C_{p}(T)$$

where $C_{p}^{0}$ refers to the heat capacity without the contribution from point defects and $\Delta C_{p}$ is the excess heat capacity due to the formation of anti‐Frenkel defects.

At each temperature, three MD simulations with different initial seeds were performed, and cubic splines were fitted to the entire set of data. Differentiating the splines yielded the heat capacity at constant pressure, $C_{p}$, according to

(8) $$C_{p}(T)={\left(\frac{\partial H_{\mathrm{tot}}}{\partial T}\right)}_{p}\approx {\left(\frac{\partial E_{\mathrm{tot}}}{\partial T}\right)}_{p}$$

The approximation in Equation (8) —replacing the derivative of $H_{\mathrm{tot}}$ with the derivative of $E_{\mathrm{tot}}$—is reasonably for the conditions (p=0) of the simulations. The calculated heat capacity of the OP phase is shown in Figure 3a. $C_{p}$ is seen to increase with increasing temperature, and for the lower‐temperature regime (950≤T/K≤2300) it was described well with a second‐order polynomial to extract $C_{p}^{0}(T)$. Extrapolating this polynomial to higher temperatures, we found an increasing difference between the $C_{p}$ data and the $C_{p}^{0}(T)$ extrapolation, i.e., $\Delta C_{p}(T)$.

**FIGURE 3:**
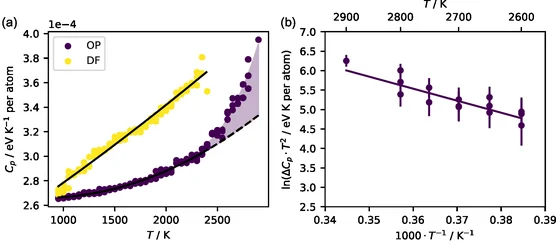
(a) Heat capacity at constant pressure, $C_{p}$, of lanthanum cerate calculated from MD simulation data [OP: ordered pyrochlore, La_2_Ce_2_O_7_; and DF: defect fluorite, (La_0.5_Ce_0.5_)O_1.75_]. The dashed line refers to the extrapolation of $C_{p}^{0}(T)$ to higher temperatures. The shaded area indicates $\Delta C_{p}$ arising from the formation of anti‐Frenkel defects. (b) Analysis of $\Delta C_{p}$ of La_2_Ce_2_O_7_ to obtain the enthalpy and entropy of anti‐Frenkel disorder, $\Delta H_{\mathrm{aF}}^{\text{⦵}}$ and $\Delta S_{\mathrm{aF}}^{\text{⦵}}$.

In order to obtain from these data the entropy and enthalpy of anti‐Frenkel disorder ($\Delta S_{\mathrm{aF}}^{\text{⦵}}$ and $\Delta H_{\mathrm{aF}}^{\text{⦵}}$), one needs to relate these quantities to $\Delta C_{p}$. One starts with the equilibrium constant for anti‐Frenkel disorder (Equation (6))

(9) $$K_{\mathrm{aF}}=\frac{c_{i}c_{v}}{c_{\mathrm{is}}c_{O}}=\exp\;\left(\frac{\Delta S_{\mathrm{aF}}^{\text{⦵}}}{k_{B}}\right)\exp\;\left(-\frac{\Delta H_{\mathrm{aF}}^{\text{⦵}}}{k_{B}T}\right)$$

where $c_{i}$ is the concentration of oxide ions on interstitial sites, $c_{v}$ is the concentration of oxygen vacancies, $c_{\mathrm{is}}$ is the concentration of interstitial sites, and $c_{O}$ is the concentration of oxide ions in the lattice. Following charge neutrality, the concentrations of both defects (interstitials and vacancies) are equal, that is, $c_{\mathrm{def}}\equiv c_{v}=c_{i}$, from which, with Equation (9), one obtains

(10) $$c_{\mathrm{def}}=\sqrt{c_{O}c_{\mathrm{is}}}\exp\;\left(\frac{\Delta S_{\mathrm{aF}}^{\text{⦵}}}{2k_{B}}\right)\exp\;\left(-\frac{\Delta H_{\mathrm{aF}}^{\text{⦵}}}{2k_{B}T}\right)$$

Expressing $\Delta C_{p}(T)$ in terms of the enthalpic contribution from each pair of defects, one finds

(11) $$\Delta C_{p}(T)=\frac{\partial \Delta H_{\mathrm{def}}}{\partial T}=\frac{\partial }{\partial T}\left(N_{O}\frac{c_{\mathrm{def}}}{c_{O}}\Delta H_{\mathrm{aF}}^{\text{⦵}}\right)$$

where $N_{O}$ is the number of oxygen ions in the simulation cell. Using Equation (10), we thus arrive at

(12) $$\Delta C_{p}(T)=N_{O}\sqrt{\frac{c_{\mathrm{is}}}{c_{O}}}\frac{{\Delta H_{\mathrm{aF}}^{\text{⦵}}}^{2}}{2k_{B}T^{2}}\exp\;\left(\frac{\Delta S_{\mathrm{aF}}^{\text{⦵}}}{2k_{B}}-\frac{\Delta H_{\mathrm{aF}}^{\text{⦵}}}{2k_{B}T}\right)$$

Consequently, a plot of $\ln\;(\Delta C_{p}\cdot T^{2})$ versus 1/T (Figure 3b) yields the required quantities from a linear fit. In this way, the standard enthalpy $\Delta H_{\mathrm{aF}}^{\text{⦵}}=(5.4\pm 1.0)\;\mathrm{eV}$ and the standard entropy $\Delta S_{\mathrm{aF}}^{\text{⦵}}=(12\pm 5)\;k_{B}$ of anti‐Frenkel disorder in the OP structure were determined.

For the DF phase, in contrast, $C_{p}(T)$ exhibits an increase with increasing temperature that can be described with a second‐order polynomial for the entire temperature range considered (Figure 3a). That is, there is no anomalous increase observed at higher temperatures. Although anti‐Frenkel disorder is known for ${\mathrm{CeO}}_{2}$ [52, 53, 54, 55], the DF phase corresponds to a high level of acceptor substitution with La. This level of substitution will shift the temperature at which this intrinsic disorder reaction will become apparent to higher temperatures (possibly even beyond the melting temperature). Hence, the results of Figure 3a for the DF phase are consistent with expectations. Comparing $C_{p}$ for the OP and DF phases, one perceives an increasing difference with increasing temperature. We tentatively ascribe this behavior to the increasing amount of disorder on the oxygen sublattice of the simulated DF phase with increasing temperature. More generally, the results of Figure 3a appear to indicate whether a composition can be classed as an OP or as a DF phase: the former, but not the latter, is expected to show an anomalous increase in $C_{p}$. With this information, we now turn to compositions with intermediate degrees of cation disorder.

### 2.3 Intermediate Compositions: $\Delta_{\mathrm{trs}}G$ and $C_{p}$

In experiment, the cation distribution in the oxygen‐deficient DF phase is not expected to be entirely random. Some degree of cation ordering will presumably occur at the high temperatures required for synthesis and sintering since cation mobility, which is needed for cations to redistribute, only becomes appreciable at such temperatures. In this section, we consider, first $\Delta_{\mathrm{trs}}G$ and then $C_{p}$, for compositions with intermediate degrees of cation disorder.

Intermediate compositions were produced from the OP phase by switching at random a given amount of cations, $N_{\mathrm{switched}}$, from one cation sublattice to the other. Relative to the total amount of cations of one species, $N_{\mathrm{total}}$, one can thus calculate a disorder parameter χ, as introduced by Perriot and Uberuaga [37], in order to describe the degree of cation disorder:

(13) $$\chi =2\cdot \frac{N_{\mathrm{switched}}}{N_{\mathrm{total}}}$$

Zero switches indicates the OP structure, with *χ* = 0; while switching the maximum amount of 50% of the cations results in the DF structure, with χ=1.0.

Lattice energies were obtained as a function of temperature by MD simulations, as described above, and thus values of $\Delta_{\mathrm{trs}}E$ were calculated according to Equation (2) for the reaction of the OP phase to structures with different degrees of cation disorder, specifically, χ=[0.2,0.4,0.6,0.8,1.0]. Only data for χ=0.2 and χ=1.0 are plotted in Figure 4a, since the other data fall between these two extremes.

**FIGURE 4:**
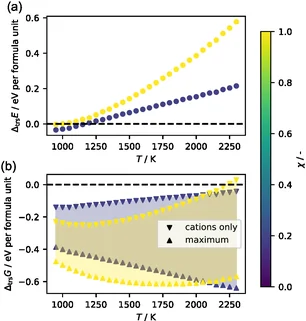
Transition from the ordered pyrochlore La_2_Ce_2_O_7_ to LCO structures with differing amounts of cation disorder χ. Only data for χ=0.2 and χ=1.0 are shown for clarity, with the data for the intermediate disordered structures lying in‐between: (a) transition energy $\Delta_{\mathrm{trs}}E$ obtained from MD simulations; (b) Gibbs transition energy $\Delta_{\mathrm{trs}}G$ obtained from $\Delta_{\mathrm{trs}}E$ and contributions from configurational entropy. The shaded areas refer to the range of $\Delta_{\mathrm{trs}}G$ for intermediate degrees of oxygen‐vacancy ordering. The dashed black line indicates a (Gibbs) energy difference of zero.

The general behavior for each structure follows closely that observed for the DF phase in Figure 2a. $\Delta_{\mathrm{trs}}E$ increases with increasing T, and it is positive, except at the lowest temperatures examined. Isothermal values of $\Delta_{\mathrm{trs}}E$ also increase with increasing χ. For the entirely disordered system (χ=1.0), the data are essentially the same as those shown in Figure ‍2a, confirming that two different procedures (I: substitution of ${\mathrm{CeO}}_{2}$ with La_2_O_3_, II: disordering of La_2_Ce_2_O_7_) produce similar results.

To obtain $\Delta_{\mathrm{cfg}}S$ and thus $\Delta_{\mathrm{trs}}G$, we used the same procedure as before, considering for $\Delta_{\mathrm{cfg}}S_{\mathrm{an}}$ the two extremum values and for $\Delta_{\mathrm{cfg}}S_{\mathrm{cat}}$, setting $S_{\mathrm{cfg},\mathrm{cat}}=0$ for χ=0 (OP structure) and extending Equation (4) to

(14) $$S_{\mathrm{cfg},\mathrm{cat}}(\chi )=-k_{B}\cdot 4\cdot \left[\frac{\chi }{2}\ln\;\frac{\chi }{2}+\left(1-\frac{\chi }{2}\right)\ln\;\left(1-\frac{\chi }{2}\right)\right]$$

for a system with two separate cation sublattices for the two species made up of the same amount of particle sites, where an amount χ/2 is switched and randomly distributed on the other sublattice. The plot for χ=1.0 in Figure 4b is, of course, very similar in shape to the plot in Figure 2b.

Although the $\Delta_{\mathrm{trs}}G$ values for χ=1.0 are lower than those for χ=0.2 for a given degree of anion disorder (i.e., for given $\Delta_{\mathrm{cfg}}S_{\mathrm{an}}$), it is not possible to ascertain which disordered structure is the most stable at a given temperature because the degree of anion disorder will vary with the degree of cation disorder χ. Nevertheless, we expect quite generally $\Delta_{\mathrm{cfg}}S_{\mathrm{an}}$ to increase with χ, so that the structures with higher cation and anion disorder will be favored. It is in any case clear that 20% cation disorder is already sufficient to make that (slightly) disordered structure more stable than the completely OP structure.

In general, this result—$\Delta_{\mathrm{trs}}G<0$ for χ>0—accords well with many XRD [3, 4, 5, 6, 7, 9, 10] and NPD [12, 14, 15] reports in the literature that favor a DF structure over the OP structure. It is also worth noting that our results of $\Delta_{\mathrm{trs}}E>0$ for χ>0 agree well with results from other simulations, which favor the OP structure [14, 18, 19, 20]. We thus show that, by including configurational entropy and considering the Gibbs energy, we are able to resolve the discrepancy between experimental and computational data.

Values of $C_{p}$ obtained across the OP–DF series are plotted in Figure 5. For higher values of χ, that is, for χ≥0.6, $C_{p}(T)$ can be described with a second‐order polynomial for the entire temperature range considered. For χ≤0.4, in contrast, the anomalous increase at higher temperatures, indicative of anti‐Frenkel disorder, is apparent. In all three cases χ=[0,0.2,0.4], the anomalous increase begins at *T* ≈ 2400 K, but the magnitude of $\Delta C_{p}$ decreases with increasing χ. This means that the scatter in the data becomes larger with increasing χ, preventing the extraction of $\Delta H_{\mathrm{aF}}^{\text{⦵}}$ and $\Delta S_{\mathrm{aF}}^{\text{⦵}}$ for all but the OP phase (data for χ=0.2 are shown in Figure 5b for illustration, but no data are shown for χ=0.4 as the error bars are even larger).

**FIGURE 5:**
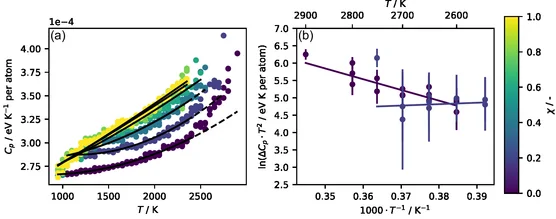
(a) Heat capacity $C_{p}$ at constant pressure of LCO structures with different degrees of cation disorder χ. The dashed lines indicate the extrapolation of $C_{p}^{0}(T)$ to higher temperatures. Differences between data and the dashed lines give the excess heat capacity due to the formation of anti‐Frenkel defects, $\Delta C_{p}$. (b) Analysis of $\Delta C_{p}$ to obtain $\Delta H_{\mathrm{aF}}^{\text{⦵}}$ and $\Delta S_{\mathrm{aF}}^{\text{⦵}}$.

Although the appearance of $\Delta C_{p}$ provides a characteristic difference between OP and DF structures, it is questionable whether this feature can be utilized experimentally. Not only are data for a huge range of temperatures required so that both $C_{p}^{0}(T)$ and $\Delta C_{p}(T)$ are both evident, but more importantly, data for systems with OP and DF behavior are also required as reference. Instead, the absolute magnitude of $C_{p}$ could be utilized, since isothermal values of $C_{p}$ at *T* = 2000 K, for example, increase with increasing χ. Highly accurate experimental data would be necessary, however. The only values available at present in the literature are in the range of (2.6 to 2.9) × 10^−4^ eV K^−1^ per atom for 673≤T/K≤1273 [56], which are consistent with our data but vary too much to allow a conclusion as to whether the sample examined experimentally had a DF‐like or OP‐like structure.

Such a comparison presupposes, of course, that the computational data of Figure 5a are accurate, and at present, there is no means of independent validation. The absolute magnitude, as noted above, is consistent with experimental data [56]. And while our $C_{p}$ data do exhibit clear trends with temperature and degree of cation disorder, with only a small amount of scatter, this essentially indicates that the data are precise, not that they are accurate. Thus, the use of the absolute magnitude of $C_{p}$ to reveal the degree of cation ordering will best be carried out having validated the computational data (e.g., with data from MD simulations with machine‐learning interatomic potentials). New, highly accurate experimental data for samples with different χ would also be highly advantageous.

### 2.4 Oxygen Tracer Diffusion

A different measure of the point‐defect behavior in LCO materials with different χ, and one that is obtained with comparative ease from MD simulations, is the oxygen tracer diffusion coefficient. This is because, in the case of oxygen diffusion occurring by a vacancy mechanism, for example, the tracer diffusion coefficient of oxygen can be expressed as

(15) $$D_{O}^{*}=f_{v}^{*}D_{v}\frac{c_{v}}{c_{O}}$$

that is, in terms of the tracer correlation coefficient $f_{v}^{*}$, the oxygen‐vacancy diffusion coefficient $D_{v}$, and the concentrations of oxygen vacancies and oxide ions, $c_{v}$ and $c_{O}$. An analogous expression holds if oxygen interstitials are solely responsible for oxygen diffusion. However, if both oxygen vacancies and oxygen interstitials contribute significantly to oxygen tracer diffusion, and if the vacancies and interstitials encounter each other frequently, as is the case in an OP phase with anti‐Frenkel disorder (for a counterexample, see Ref. [57]), $D_{O}^{*}$ is not simply given by the sum of the contributions [58]. We thus expect complicated behavior for the OP‐like systems and relatively simple behavior for the DF‐type systems. The ionic mobility does not suffer from this problem [34], but the direct calculation of ionic mobilities from MD simulations is a substantial undertaking, as data as a function of temperature and electric field are required in order to be certain of obtaining data that refer to the linear‐response regime. This subject is left for future study.

The tracer diffusion coefficient of oxygen was obtained from an MD simulation by monitoring the change in the mean squared displacement (MSD) of oxide ions, $\left\langle r_{O}^{2}\right\rangle$, as a function of time t. If linear behavior is observed, then $D_{O}^{*}$ is given by

(16) $$D_{O}^{*}=\frac{1}{6}\frac{d\langle r_{O}^{2}\rangle }{dt}$$

While $\left\langle r_{O}^{2}\right\rangle$ data may at first sight appear linear, it is important to confirm that the behavior is diffusive rather than subdiffusive [47], and this is done by considering the α‐value (see Section 4). Another important factor to consider is whether the jump statistics are sufficient to allow a reliable determination of the tracer diffusion coefficient. For this purpose, the relative error in the tracer diffusion coefficient was calculated from

(17) $$u_{\mathrm{rel}}=\sqrt{\frac{2}{d\cdot N_{O}\cdot \left[1-\exp\;\left(-\frac{{\left\langle r_{O}^{2}\right\rangle }_{f}}{s_{O}^{2}}\right)\right]}}$$

where d is the number of dimensions in which diffusion occurs, $N_{O}$ is the number of oxide ions in the simulation cell, ${\left\langle r_{O}^{2}\right\rangle }_{f}$ is the final value of the oxide ions’ MSD, and $s_{O}$ is the jump distance of an oxide ion [59]. Results for $D_{O}^{*}$ are plotted in Figure 6.

**FIGURE 6:**
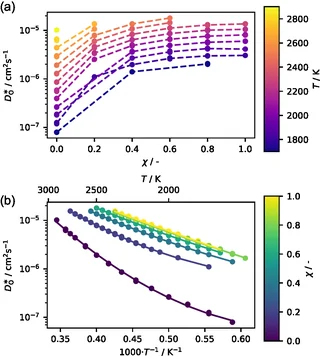
Tracer diffusion coefficient of oxygen in LCO structures (a) as a function of the disorder parameter χ for a temperature range of 1700≤T/K≤2900 (the dashed lines are guides to the eye) and (b) as a function of inverse temperature for 0≤χ≤1 (solid lines are second‐order polynomial fits). Error bars are smaller than the symbol size.

In Figure 6a, one sees that, at a given temperature, $D_{O}^{*}$ increases strongly from χ=0 to χ=0.2; it increases weakly from χ=0.2 to χ=0.8; and it ceases to increase from χ=0.8 to χ=1.0. That is, 20% cation disorder is already sufficient to increase the oxygen tracer diffusivity by orders of magnitude, as found previously for ${\mathrm{Gd}}_{2}{\mathrm{Ti}}_{2}O_{7}$ and ${\mathrm{Gd}}_{2}{\mathrm{Zr}}_{2}O_{7}$ in accelerated MD studies by Perriot and Uberuaga [37]. And taking the isothermal behavior of $D_{O}^{*}$ as a measure, one could claim that all the structures—with the exception of the OP structure—could be classified as DF‐like structures.

The main result from the Arrhenius plot of Figure 6b is that the structures with low χ exhibit pronounced curvature, while those with high χ show close to linear behavior. Determining an activation enthalpy would be inappropriate for the former since linear fits would yield poor descriptions. Rather than considering this a problem, we made use of it, obtaining another quantitative measure to differentiate between OP‐like and DF‐like behavior. Specifically, we determined a temperature‐dependent activation enthalpy of oxygen diffusion, $\Delta H_{D^{*}}(T)$, by fitting a second‐order polynomial to the data and evaluating the polynomial according to

(18) $$\Delta H_{D^{*}}(T)=-k_{B}\frac{d\ln\;D_{O}^{*}}{d(1/T)}$$

The results are shown in Figure 7 for the temperature range for which data are available for all structures, 1800≤T/K≤2400. The range of activation enthalpies displayed by a particular structure decreases with increasing χ, but the general trend is gradual, making it difficult to identify where the boundary between OP and DF lies. Clearly, the large variations for χ=0 and χ=0.2 indicate OP‐like behavior, while the almost constant values obtained for χ=0.8 and χ=1.0 indicate DF‐like behavior. For the two intermediate compositions, $\Delta H_{D^{*}}$ varies significantly, but by a far smaller amount than those seen for the more ordered structures, and one could equally well say that these intermediate compositions belong to the OP or to the DF group.

**FIGURE 7:**
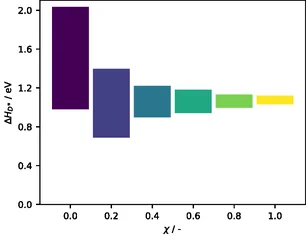
Temperature‐dependent activation enthalpies of oxygen tracer diffusion, $\Delta H_{D^{*}}$, in LCO as a function of the disorder parameter χ obtained from the data in Figure 6 for 1800≤T/K≤2400.

The curvature observed for the more ordered structures in the Arrhenius plots of Figure 6b is attributed to two effects: two differently mobile oxygen defects that encounter each other frequently (see also Section 2.5) and local, transient jump processes, particularly at lower temperatures. Examination of ion trajectories in MD runs at lower temperatures revealed predominantly a four‐jump process involving three oxide ions, with the four jump vectors forming a square: an oxide ion on a 48f site jumps to an 8a site to create a vacant 48f site, two further oxide ions jump so as to move the vacancy next to the occupied 8a site, and finally the first oxide ion jumps into this vacancy to return the lattice to its initial (perfect) state (but with three shifted oxide ions). This does not constitute long‐range diffusion, but it obviously contributes to the oxide‐ions’ MSD, and hence yields a nonzero value for $D_{O}^{*}$. For the DF‐like structures, both effects are absent, and this accounts, then, for $D_{O}^{*}$ displaying no significant curvature but linear behavior.

### 2.5 Interstitial and Vacancy Diffusion in the OP Structure

In order to examine separately the behavior of oxygen interstitials and oxygen vacancies in the OP structure, oxygen tracer diffusion coefficients were obtained from OP cells in which either oxide ions had been removed (from the 48f sites) to introduce oxygen vacancies or oxide ions had been added (to the 8a sites) to introduce oxygen interstitials. Three different levels of defects were introduced in each case, and the results are plotted in Figure 8a together with data for the stoichiometric OP structure. In both cases—oxygen vacancies and oxygen interstitials—the tracer diffusion coefficient of oxygen increases upon the introduction of additional defects, with the increase becoming larger with increasing defect concentration. In addition, added vacancies are seen to cause a larger effect than added interstitials, implying that vacancies are more mobile than interstitials. All data exhibit significant curvature in the Arrhenius plots.

**FIGURE 8:**
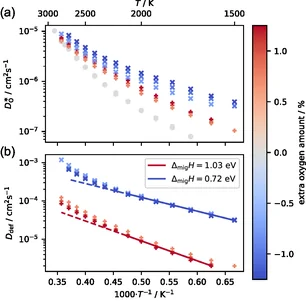
(a) Oxygen tracer diffusion coefficients $D_{O}^{*}$ in La_2_Ce_2_O_7*y*_ as a function of the inverse temperature for ordered pyrochlore cells with 0.75%, 1.00%, and 1.25% oxygen interstitials (y=[1.0075,1.01,1.0125]) or with 0.75%, 1.00%, and 1.25% oxygen vacancies (y=[0.9925,0.99,0.9875]), in comparison with data for the stoichiometric cell (y=1); (b) Defect diffusion coefficients $D_{\mathrm{def}}$ calculated from $D_{O}^{*}$ data for different defect concentrations, with solid lines indicating the data used for linear regression and dashed lines the extrapolations.

Given the significant increases, it can be assumed that only one type of defect determines $D_{O}^{*}$, and hence that Equation (15) (and its equivalent form for interstitials) can be used to extract defect diffusion coefficients (if tracer correlation coefficients are ignored). In this way, the oxygen‐vacancy and oxygen‐interstitial diffusion coefficients, $D_{v}$ and $D_{i}$, shown in Figure 8b are obtained. At lower temperatures, the data collapse onto single lines, as they should for dilute solutions of defects for which Equation (15) is valid. Oxygen vacancies are thus found to be more mobile than oxygen interstitials, confirming the conclusion reached above, and the respective activation enthalpies of defect migration (obtained through linear regression of the three data sets for 1500≤T/K≤2100) are Δ_mig_ *H*_v_ = 0.72 eV and Δ_mig_ *H*_i_ = 1.03 eV. At higher temperatures (*T* > 2100 K), the calculated $D_{v}$ and *D*_i_ values deviate from the extrapolated linear behavior, and this is due to the number of defects generated by the intrinsic defect reaction (anti‐Frenkel disorder) becoming increasingly important.

In the previous section, we emphasized that, in the case of vacancy and interstitial migration contributing to tracer diffusion in the OP structure, the tracer diffusion coefficient cannot be expressed as the simple sum of the two individual contributions. If it were possible, $D_{O}^{*}$ could be predicted precisely for the OP structure from

(19) $$D_{O}^{*}=\frac{c_{\mathrm{def}}}{c_{O}}\left(D_{i}+D_{v}\right)$$

since $D_{v}$ and *D*_i_ values are available from Figure 8b and since $c_{v}=c_{i}=c_{\mathrm{def}}$ can be calculated from Equation (10) with the entropy and enthalpy of anti‐Frenkel disorder from the $C_{p}$ analysis. Figure 9 shows the comparison of $D_{O}^{*}$ values predicted with Equation (19) and $D_{O}^{*}$ values extracted from the analysis of $\left\langle r_{O}^{2}\right\rangle$. The substantial underestimation of $D_{O}^{*}$ by the prediction confirms quantitatively that Equation (19) is not valid for oxygen tracer diffusion in the OP structure when both vacancy and interstitial migration are active, and the defects encounter each other frequently [58].

**FIGURE 9:**
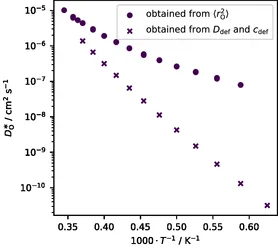
Oxygen tracer diffusion coefficients in the OP structure of LCO, calculated from the mean squared displacements ($\left\langle r_{O}^{2}\right\rangle$) or from Equation (19) with defect diffusion coefficients $D_{\mathrm{def}}$ and defect concentrations $c_{\mathrm{def}}$.

### 2.6 Comparison With Experiment

Oxygen tracer diffusion coefficients have not been measured experimentally for LCO, but electrical conductivities have. One can convert an oxygen tracer diffusion coefficient into an oxide‐ion conductivity with the Nernst–Einstein equation (with electronic charge e and oxide‐ion charge number $z_{O}$ [60]),

(20) $$\sigma_{\mathrm{def}}=\frac{e^{2}}{k_{B}T}z_{O}^{2}D_{O}^{*}c_{O}$$

if tracer and conductivity correlation factors are ignored. We may, however, only convert $D_{O}^{*}$ data for DF‐like structures because such data are not affected by the presence of two mobile defects that often encounter each other and local, transient processes [58]. Given the discussions in the previous sections regarding $D_{O}^{*}$ and $\Delta H_{D^{*}}$, we converted data for χ≥0.4 (since data for χ≤0.2 refer most probably to OP structures). In making the comparison, one needs to keep in mind that a measured conductivity may contain contributions from all mobile charge carriers, and in this case, that could be oxide ions and protons, as well as electronic charge carriers. For this reason, we include only data that, in our opinion, refers to oxide‐ion conduction (dry conditions, conductivity independent of oxygen partial pressure). The comparison with literature data [13, 22, 23, 24, 39] is made in Figure 10.

**FIGURE 10:**
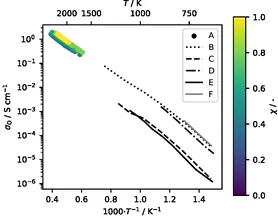
Comparison of calculated and reported experimental oxide‐ion conductivities for LCO: A—this study, calculated from $D_{O}^{*}$ data for LCO structures with χ≥0.4; B—Besikiotis et al. [22]; C—Mowe et al. [13]; D—Sanjuán et al. [23]; E—Zhang et al. [24]; and F—Zamudio‐García et al. [39].

The literature data in Figure 10 show a substantial discrepancy (by more than an order of magnitude) in the isothermal values of the oxide‐ion conductivity $\sigma_{O}$. The origin of this discrepancy is unclear. Good quantitative agreement is seen between data from three studies [22, 23, 39] and our calculated $\sigma_{O}$ for high χ, extrapolated to lower temperatures. For all literature studies [13, 22, 23, 24, 39], the activation enthalpy of oxide‐ion conduction is (1.0 to 1.1) eV, and these values agree very well with our values calculated for the systems with high χ (see Table 1). Although we do not show data for the OP structure, we can confirm that the experimental data is inconsistent with an OP structure in which anti‐Frenkel disorder governs the anion defect concentrations. The activation enthalpy of oxide‐ion conduction in the case of predominantly vacancy migration (expected for lower temperatures) would be $\Delta_{\mathrm{mig}}H_{v}+\Delta H_{\mathrm{aF}}^{\text{⦵}}/2\approx 3.4$ eV, and thus far in excess of experimental data.

**TABLE 1:** Activation enthalpy of oxide‐ion conduction $E_{a}$ in LCO. The values given for this study refer to the minimum values shown in Figure 7 (which thus refer to MD data for the lowest temperatures examined).

Source	χ, –	$E_{a}$, eV
This study	1.0	1.03
This study	0.8	0.99
This study	0.6	0.94
This study	0.4	0.90
Besikiotis et al. [22]		1.12
Mowe et al. [13]		1.01
Sanjuán et al. [23]		1.08
Zhang et al. [24]		1.11
Zamudio‐García et al. [39]		1.12

## 3 Conclusions

Selected thermodynamic and kinetic properties of LCO were studied with atomistic simulations, with the two extreme structure types being the OP and the disordered DF structures. There are three results to be highlighted.

1. The tendency of an oxide to form a DF rather than an OP is widely considered in the literature in terms of the formation of cation antisite defects and anti‐Frenkel defects [38, 61, 62]. Here, we took a different approach, determining first the transition energy from the ordered pyrochlore to variously disordered structures and, subsequently, through inclusion of configurational entropy, the Gibbs transition energy. Our results clearly indicate that the disordered structures are thermodynamically more stable. It was not possible, however, to determine which disordered structure is the most stable. Given the importance of entropy, we surmise that this will depend on temperature, opening up the possibility of generating LCO structures with different degrees of cation disorder through thermal annealing at different temperatures.
2. Determining the isobaric heat capacity and the oxygen tracer diffusivity as a function of temperature for variously disordered structures, we found behavioral differences between OP‐like and DF‐like structures. The heat capacity exhibited an additional increase at high temperatures for OP‐like structures owing to the formation of anti‐Frenkel defects but not for DF‐like structures. The oxygen tracer diffusivity displayed in Arrhenius plots of the range of temperatures considered substantial curvature for OP‐like structures but not for DF‐like structures, and this behavior was linked to the types of defects present and migration mechanisms. The boundary between OP‐like and DF‐like structures, however, was not unique—different properties yielded different answers.
3. Good agreement is demonstrated between our MD data for disordered structures and some experimental oxide‐ion conductivities in terms of the absolute magnitude of the conductivity and with all experimental data in terms of the activation enthalpy of conduction. This result thus constitutes further evidence that experimental structures are DF‐like.

## 4 Computational

The MD simulations were carried out with the lammps code [63] with a 6×6×6 (pyrochlore) or 12×12×12 (fluorite) supercell (containing 19,008 particles for the stoichiometric cell) and periodic boundary conditions in all dimensions. Particle number N, pressure p and temperature T were kept constant (NpT ensemble) using a Nosé–Hoover thermostat (barostat) [64, 65] with a damping parameter of 0.1 ps (1 ps). A pressure of 1 bar was used in all simulations. The time step used in the simulations was 1 fs, and Newton’s equation of motion was integrated with the velocity Verlet algorithm. The long‐range coulombic interactions were calculated with a particle–particle–particle–mesh solver with an accuracy of $10^{-5}$.

The short‐range interactions were modeled with the CRG many‐body potential [44]. The potential energy $U_{j}$ of an atom j is calculated as

(21) $$U_{j}=\frac{1}{2}\sum_{k}\varphi_{\alpha \beta }(r_{jk})-G_{\alpha }\sqrt{\sum_{k}\sigma_{\beta }(r_{jk})}$$

The potential is based on the embedded atom method [66] containing two terms: the first includes a sum of Morse and Buckingham potentials

(22) $$\varphi_{\alpha \beta }(r_{jk})=\varphi_{M}(r_{jk})+\varphi_{B}(r_{jk})$$

with the former being defined as

(23) $$\begin{array}{cc} \varphi_{M}(r_{jk}) & =D_{\alpha \beta }\{\exp\;\left[-2\gamma_{\alpha \beta }(r_{jk}-r_{0})\right]\\ & \;-2\exp\;\left[-\gamma_{\alpha \beta }(r_{jk}-r_{0})\right]\} \end{array}$$

and the latter as

(24) $$\varphi_{B}(r_{jk})=A_{\alpha \beta }\exp\;\left(\frac{-r_{jk}}{\rho_{\alpha \beta }}\right)-\frac{C_{\alpha \beta }}{r_{jk}^{6}}$$

The simple pair interactions between two particles j and k separated by a distance $r_{jk}$ are described. The second term is a nonlinear embedding function including the pairwise interaction $\sigma_{\beta }(r_{jk})$ as

(25) $$\sigma_{\beta }(r_{jk})=\frac{n_{\beta }}{r_{jk}^{8}}$$

The term is used to calculate the energy required to introduce an atom j of type α into the electron cloud distribution of the surrounding atoms of type β. The potential parameters used for LCO are shown in Table 2 and are a combination of two parameter sets. The parameters, including Ce and O, are taken from the original work introducing the CRG potential, describing the use for ${\mathrm{CeO}}_{2}$ doped with different actinides [44]. The La—O interaction is derived in a subsequent work [45]. The charge numbers of the atom types are $z_{\mathrm{La}}=+1.6656$, $z_{\mathrm{Ce}}=+2.2208$, and $z_{O}=-1.1104$. The cutoff of the short‐range interaction was set to 11.0 Å.

**TABLE 2:** Potential parameters for the many‐body potential used in this study [44, 45].

α–β	La─O	Ce─O	O─O	Ce─Ce
$D_{\alpha \beta }$, eV		0.7193		
$\gamma_{\alpha \beta }$, Å^−1^		1.869		
$r_{0}$, Å		2.356		
$A_{\alpha \beta }$, eV	36,197.135	351.341	830.283	18,600
$\rho_{\alpha \beta }$, Å	0.2069536	0.3805	0.3529	0.2664
$C_{\alpha \beta }$, eV Å^6^	0.000	0.0	3.8843	0.0
$G_{\alpha }$, eV Å^1.5^			0.69	0.308
$n_{\beta }$, Å^5^			106.856	1556.803

For systems with substantial disorder on the oxygen sublattice, a MMC run was carried out before the MD production run at *T* = 2000 K with 500 attempted swaps of oxide ions and oxygen vacancies for all 1000 MMC steps. After that an optimization of the particle positions and box sizes with tolerances of 10^−25^ for the energy and 10^−25^ eV/Å for the forces was carried out. The MD equilibration and production runs were not kept in a constant time frame for the whole temperature range of up to 2800 K. If the equilibration time is sufficient, the data were evaluated after every few hundred picoseconds by means of the α‐value defined in

(26) $$\langle r_{O}{\left(t\right)}^{2}\rangle \propto {\left(t-t_{0}\right)}^{\alpha }$$

To calculate a diffusion coefficient from the mean square displacement $\left\langle r_{O}^{2}\right\rangle$ as a function of simulation time t, an α‐value of 1.0 is required, indicating diffusive behavior [47]. To make sure the equilibration time $t_{0}$ is long enough, the minimum value for α was set to 0.9. If this was not reached, the calculation was extended. Example data of $\left\langle r_{O}^{2}\right\rangle$ and α are plotted in Figure ‍11. As is evident, lower temperatures require longer equilibration times.

**FIGURE 11:**
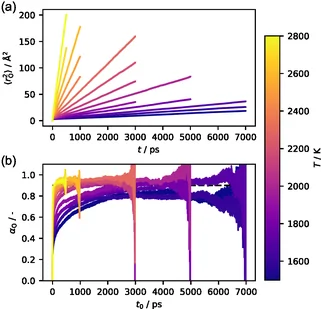
(a) Mean square displacement $\left\langle r_{O}^{2}\right\rangle$ of oxygen in lanthanum cerate with disorder parameter χ=1.0 as a function of calculation time t for a temperature range of *T *= 1000–3000 K, as well as (b) α value from Equation (26) .

## Funding

This study was supported by Deutsche Forschungsgemeinschaft (Grant 523164409).

## Conflicts of Interest

The authors declare no conflicts of interest.

## Data Availability

The data that support the findings of this study are available from the corresponding author upon reasonable request.
